# A Multicenter, Randomized, Double-Blind, Placebo-Controlled Study of Compound Glycyrrhizin Capsules Combined with a Topical Corticosteroid in Adults with Chronic Eczema

**DOI:** 10.1155/2020/6127327

**Published:** 2020-03-30

**Authors:** Wei Xu, Yan Li, Mei Ju, Wei Lai, Xueyan Lu, Huijuan Shi, Weimin Shi, Heng Gu, Linfeng Li

**Affiliations:** ^1^Department of Dermatology, Beijing Friendship Hospital, Capital Medical University, Beijing, China; ^2^Chinese Academy of Medical Sciences, Peking Union Medical College, Nanjing, Jiangsu, China; ^3^Department of Dermatology, The Third Affiliated Hospital, Sun Yat-Sen University, Guangdong, Guangzhou, China; ^4^Department of Dermatology, Peking University Third Hospital, Beijing, China; ^5^Department of Dermatology, General Hospital of Ningxia Medical University, Ningxia, Yinchuan, China; ^6^Department of Dermatology, Shanghai General Hospital, Shanghai, China

## Abstract

**Background:**

Glycyrrhizin is widely used in skin disorders, such as psoriasis, alopecia areata, and allergic diseases, but has not been extensively studied in patients with chronic eczema.

**Objective:**

To evaluate the efficacy and safety of oral compound glycyrrhizin (OCG) plus topical corticosteroid (TCS) in adults with chronic eczema.

**Methods:**

This was a multicenter, randomized, double-blind, placebo-controlled study in patients with chronic eczema (*n* = 199). Randomized participants from 6 centers in China received either 75 mg OCG capsules or placebo capsules three times a day and TCS (i.e., 0.1% mometasone furoate ointment) once a day for 28 days. Efficacy was determined by analyzing the mean change from the baseline using standardized measures including the Investigator's Global Assessment (IGA) score, Eczema Area and Severity Index (EASI), and the visual analogue scale score (VAS) of itching.

**Results:**

Decreases in absolute EASI were significantly greater in the OCG-treated group versus placebo on day 14 (−3.41 ± 1.41 vs. −2.71 ± 1.25, *P* < 0.001) and day 28 (−7.39 ± 1.71 vs. −6.64 ± 1.75, *P*=0.003). OCG-treated patients also saw greater benefit in other EASI metrics including EASI-50 (96.8% vs. 87.9%, *P*=0.021) and EASI-75 (47.9% vs. 21.2%, *P* < 0.001) on day 28 compared with placebo. The absolute IGA score reductions were also significantly greater in the OCG group than the placebo (all *P* < 0.05). In addition, proportions of patients who achieve clear (0) IGA scores or almost clear (1) IGA scores were significantly higher in the treated group than placebo on day 14 (22.8% vs. 6.2%, *P*=0.001) and day 28 (93.5% vs. 79.4%, *P*=0.005). Moreover, the proportions of patients with reduced pruritus were significantly greater in the treated group than placebo on day 28 (94.7% vs. 83.8%, *P*=0.016) and eczema recurrence was notably less in the OCG group versus placebo (3.19% vs. 12.12%, *P*=0.021). Eleven patients experienced adverse events, but there was no significant difference in the proportion of patients affected (3.0% vs. 8.5%, *P* > 0.05). The most common adverse events were edema of both lower limbs.

**Conclusion:**

For adults with chronic eczema, OCG capsules combined with TCS is an effective and well-tolerated treatment, suggesting that OCG may be a useful nonsteroidal agent with an additional effect for the treatment of chronic eczema by TCS.

## 1. Introduction

Eczema, known as “atopic dermatitis (AD)” and “atopic eczema” in most developed countries, is a common chronic and recurrent inflammatory skin disease [1]. In 2016, the incidence of eczema in Chinese adults was estimated to be 7.8% [2]. Eczema is highly associated with chronic pruritus, which can lead to sleeping problems, attention difficulties, social withdrawal, and a lower psychological wellbeing [3]. Topical corticosteroids (TCS) and calcineurin inhibitors are the standard first-line treatments for mild eczema [4, 5]. However, these treatments can cause adverse events such as atrophoderma and secondary infection [4, 5]. Systemic medications, including oral corticosteroids and immunosuppressants, are often used for difficult-to-treat or refractory moderate-to-severe eczema [6]. However, these treatments are not preferable due to known associations of long-term corticosteroid and immunosuppressant use with severe adverse side effects, including hypothalamic-pituitary-adrenal axis suppression, osteoporosis, hip aseptic necrosis, hypertension, altered immune function, and hepatosplenic T-cell lymphomas [7–9]. To circumvent long-term use of these medications, alternative therapies such as traditional Chinese medicine have become increasingly popular in patients with chronic eczema [10, 11].

Glycyrrhizin is widely used to treat inflammatory skin diseases, such as dermatitis, alopecia areata, psoriasis, and vitiligo [12–14]. The main ingredients of oral compound glycyrrhizin (OCG) capsules are licorice-extracted glycyrrhizin glycoside, glycine, and methionine.

Our study sought to compare the efficacy and safety of combining of OCG plus the topical cortical steroid (TCS) mometasone furoate, versus TCS alone in a Chinese adult population with chronic eczema. To our knowledge, this is the first multicenter, randomized, double-blind, placebo-controlled trial investigating the efficacy and safety of OCG to treat chronic, localized eczema.

## 2. Methods

### 2.1. Study Design

This was a multicenter, randomized, double-blind, placebo-controlled clinical trial to investigate the efficacy and safety of OCG in adults with chronic, localized eczema. This clinical trial was registered at the Chinese Clinical Trial Registry (Registration Number: ChiCTR-IPR-17011511). Patients were enrolled between October 2015 and December 2016 from six clinical centers, located in five metropolitan areas of China: Beijing, Shanghai, Guangzhou, Nanjing, and Yinchuan.

The Institutional Ethics Committee of each participating clinical center approved the study protocol. Written informed consent was obtained from each participant prior to enrollment, and the study protocol was made available upon request.

### 2.2. Patient Demographics

All the patients were in-patients. Patient inclusion criteria were as follows: (1) voluntarily sign the informed consent form and willing to follow the requirement defined in the study protocol; (2) aged 18 to 80 years; (3) with a confirmed diagnosis of chronic localized eczema according to the “Eczema Diagnosis and Treatment Guidelines (2011)” [15] recommended by the Department of Dermatology of the Chinese Medical Association Section of Dermatology; (4) skin lesion area of 5%–15% of body surface area (BSA), which was assessed using the palm method (patient's palm area was considered 1% of BSA); and (5) the severity of skin lesion was moderate or above (the Investigator's Global Assessment (IGA) score ≥3). The diagnosis according to the guidelines for chronic localized eczema was mainly based on patient medical history, rash morphology, and disease course [16]. In brief, skin lesions were characterized by erythema, pimples, infiltration, hypertrophy, mossy, scratches, and pigmentation; the disease course should be ≥3 months or recurrent eczema; patients should experience severe itching.

Patients were excluded from the study based on the following criteria: (1) systemic disease or other skin diseases that may affect drug efficacy assessment or skin lesion evaluation; (2) skin lesions limited to palms and feet; (3) infections in the index skin lesions requiring anti-infective therapy; (4) treatment with systemic glucocorticoids, immunosuppressants and/or UV irradiation therapy within the last 4 weeks, local use of glucocorticoids, and oral antihistamines drug within the last 2 weeks; (5) allergy to any of the components of the trial medications; (6) participation in other clinical trials at the time of enrollment interview; (7) alcohol or drug dependency/addiction; and (8) pregnancy, breastfeeding, or preparing for pregnancy.

Patients' baseline clinical data were recorded, including demographics, medical and medication history, blood test results, Investigator's Global Assessment (IGA) score, pruritus assessment (VAS method), Eczema Area and Severity Index (EASI) score of the skin lesion, and photos of the skin lesions.

### 2.3. Interventions

The study medication used was OCG capsules (batch no.: 15080301, Beijing Kawin Technology Co., Ltd.). Each capsule contains 25 mg glycyrrhizin, 25 mg glycine, 25 mg methionine, and 65 mg accessory components including lactose, calcium carbonate, microcrystalline cellulose, sodium carboxymethyl starch, talc, magnesium stearate, and povidone K30. The placebo capsules (Kai Yan Gan Le accessories, batch no.: 15080301) contained only the accessory components without glycyrrhizin, glycine, or methionine. The TCS used was mometasone furoate 0.1% ointment (10 g/support (10 g: 10 mg) Lot number: 15NGFA220, Bayer HealthCare Co. Ltd.).

**Figure 1:**
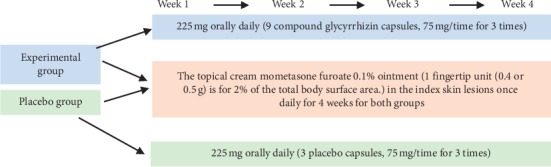
Study duration and treatment regimen. The experimental group was given 75 mg OCG capsules three times daily along with a TCS (i.e., mometasone furoate 0.1%) ointment once daily for 28 days. The control group received placebo + TCS. Relevant clinical data were collected on days 1, 7, 14, 21, and 28 and at a four-week follow-up.

Patients were randomized into two groups receiving either 75 mg of OCG or placebo three times daily for four consecutive weeks. Both groups also used the TCS at a dose of one fingertip unit for approximately 2% of the total body surface area, on the index skin lesions once daily [17]. All patients were followed for four weeks after the end of the treatment. The intervention regimen is illustrated in Figure 1. Patient visit schedule and tasks are shown in Table 1. Relevant clinical data were collected on days 1, 7, 14, and 28.

### 2.4. Efficacy Evaluation

The primary measure of efficacy was changes from baseline in EASI score. EASI score calculation method is shown in Supplementary [Supplementary-material supplementary-material-1]. We also used two additional disease severity metrics. Reduction in IGA score (Supplementary Tables [Supplementary-material supplementary-material-1]) [18] was assessed to determine treatment success rate, defined as the percentage of patients whose IGA score was reduced to 0 (clear) or 1 (almost clear) after the end of the treatment. Pruritus alleviation was assessed using the visual analogue scale score of itching (VAS) method (Supplementary Tables [Supplementary-material supplementary-material-1]) and defined as achieving none (grade 0), mild (grade 1), or ≥1 grade of pruritus reduction from baseline.

### 2.5. Safety and Recurrence Evaluation

Safety evaluations included adverse event reporting, routine physical examinations, laboratory tests, and electrocardiograms (ECG). All adverse events were recorded. Laboratory tests included blood biochemistry, urine, and stool analysis. Recurrence was defined as an ESAI score increase ≥10% from baseline in follow-up visits.

### 2.6. Sample Size

Sample size was calculated using the following parameters: the expected average EASI reduction in the OCG-treated and placebo groups based on our experience in clinical practice was 7.4 and 6.6, respectively; standard deviation was 1.8, *α* = 0.05 and *β* = 0.2 in two-tailed test. The calculated required number of patients was at least 80 for both each group. Considering a 15% dropout rate, the required number of patients for each group was estimated to be 94. Therefore, a minimum of 188 patients were planned to be enrolled.

### 2.7. Randomization

Before assigning block randomization, all participants will be informed that they will be distributed at 1 : 1 ratio to two groups: OCG-treated and placebo groups. A seed number was given to all the enrolled patients. Participants will be randomly assigned via computer-generated sequential random numbers. The statistical analysis software SAS was used to generate random numbers. A biostatistician generated the random allocation sequence. A designated study nurse enrolled participants and assigned participants to interventions by identical containers labeled with random numbers, which correspond to the participant's assigned number.

### 2.8. Blinding

The study was double-blinded. The investigation drug and placebo had an identical appearance. The study remained blinded to all individuals (including patients, investigators, and study personnel) until the time of prespecified unblinding, except for the statistician who provided the randomization sequence. The randomization list and blinding codes will be kept strictly confidential.

### 2.9. Statistical Analysis

The full analysis set (FAS) included patients taking the trial drug at least once that participated in at least one clinical visit during the course of treatment. The per-protocol set (PPS) was a subset of FAS, containing patients who filled in Case Report Form (CRF)-required contents and completed the trial with a dose of 80%–120%, good compliance, and did not use banned drugs during the trial. All primary and secondary outcome endpoints were analyzed in both the FAS and PPS sets. The Last Observation Carried Forward (LOCF) was used to analyze the missing data.

Data are presented as means ± standard deviation (SD). SAS 9.4 statistical software was used for data analysis. We used paired *t*-tests to evaluate the changes after treatment in each group. The differences between the two groups were compared using independent *t*-tests and covariance analysis (ANCOVA). The count data are presented as frequency (i.e., composition ratio). Changes in count variables were analyzed using *X*^2^ test or Fisher exact test. Changes in EASI, IGA, and VAS scores were descriptively analyzed. The safety analysis set (SAS) refers to the cases that received at least one medication and at least one safety visit. Two-sided tests were used for all statistical tests, and *P* < 0.05 was considered statistically significant.

## 3. Results

### 3.1. Patient Baseline Clinical Data

**Figure 2:**
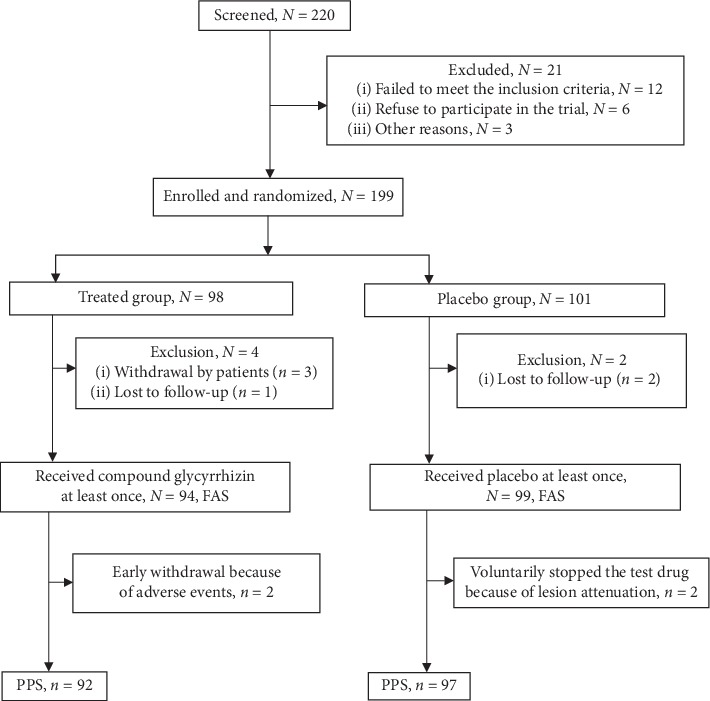
Trial schema.

Of the 220 patients enrolled, 199 were randomized to either of the two groups. Six patients discontinued the study, leaving 193 patients in the FAS (Figure 2). Of these, four participants were then excluded from the PPS because of adverse events or subsided lesions (Figure 2). Thus, the PPS included 189 patients, 92 (92/98, 93.9%) in the OCG + TCS group and 97 (97/101, 96.0%) in the placebo + TCS group. Baseline demographics and disease characteristics were similar in both groups (Table 2).

### 3.2. Efficacy Outcomes

**Figure 3:**
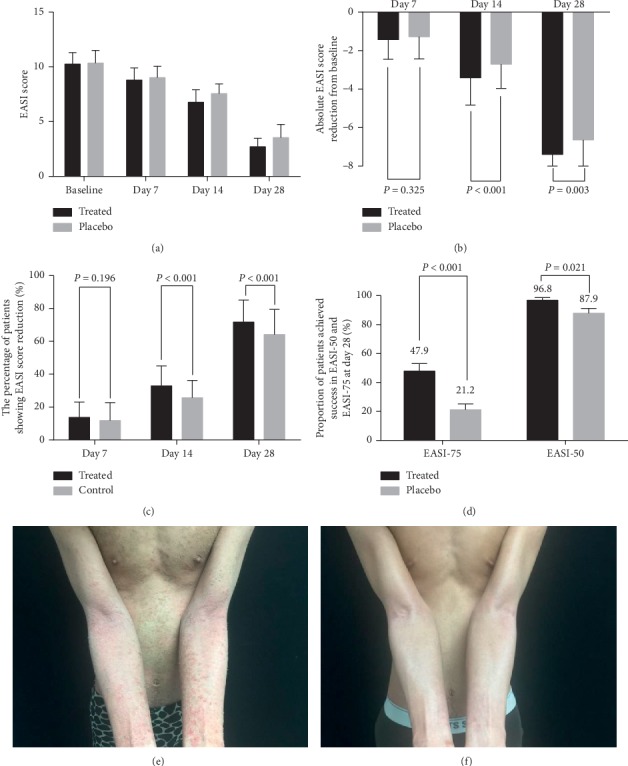
Efficacy analyses (full analysis set). (a) Comparison of mean EASI score on days 7, 14, and 28 after treatment. (b) Absolute EASI score reduction from baseline on days 14 and 28. (c) The percent of patients showing EASI reduction on days 14 and 28. (d) Proportion of patients achieving reduction in EASI-50 and EASI-75 on day 28. (e) Photographs of a patient before treatment of OCG + TCS. (f) Photographs of the patient 28 days after OCG + TCS treatment.

EASI scores continuously decreased during the trial in both groups, indicating a continuous reduction in skin lesions (Figure 3(a)). The mean absolute reduction in EASI scores from baseline in the OCG group was significantly greater than that in the placebo on both day 14 (−3.41 ± 1.41 vs. −2.71 ± 1.25, *P* < 0.001) and day 28 (−7.39 ± 1.71 vs. −6.64 ± 1.75, *P*=0.003, Figure 3(b)). *T*-tests and the ANCOVA test were both statistically significant. The percentage of participants with EASI reduction was also significantly higher in the OCG group versus placebo on both day 14 (32.86% vs. 25.67%, *P* < 0.001) and day 28 (71.73% vs. 64.16%, *P* < 0.001) (Figure 3(c)). The proportions of patients achieving success in EASI-50 and EASI-75 in the treated group on day 28 were significantly greater than those in the placebo (EASI-50: 96.8% vs. 87.9%, *χ*^2^ = 5.364, *P*=0.021 and EASI-75: 47.9% vs. 21.2%, *χ*^2^ = 15.23, *P* < 0.001, Figure 3(d)). Figures 3(e) and 3(f) show representative photos of a patient with significant EASI from day 0 (EASI = 15.2) to day 28 (EASI = 1.2).

**Figure 4:**
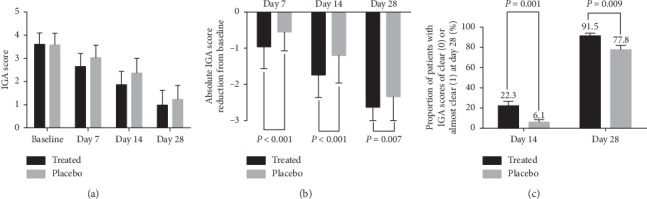
Efficacy analysis. (a) IGA scores on days 7, 14, and 28. (b) Absolute IGA score reduction from baseline on days 7, 14, and 28 was significantly greater in the OCG-treated group than in the placebo group. (c) Proportion of patients with IGA score of 0 or 1 on days 14 and 28.

On days 7, 14, and 28, the mean IGA score was reduced significantly in both groups compared with the corresponding baselines (*P* < 0.001, Figure 4(a)). The absolute reduction in IGA score from the baseline was significantly greater in the OCG group versus placebo (day 7: −0.96 ± 0.60 vs. −0.55 ± 0.52; day 14: −1.74 ± 0.62 vs. −1.20 ± 0.76; day 28: −2.63 ± 0.75 vs. −2.34 ± 0.70, all *P* < 0.05, Figure 4(b)). There was also a significantly higher proportion of patients achieving IGA scores of 0 or 1 in the OCG group compared to placebo on day 14 (22.3% vs. 6.1%, *χ*^2^ = 10.621, *P*=0.001) and day 28 (91.5% vs. 77.8%, *χ*^2^ = 6.905, *P*=0.009, Figure 4(c)).

**Figure 5:**
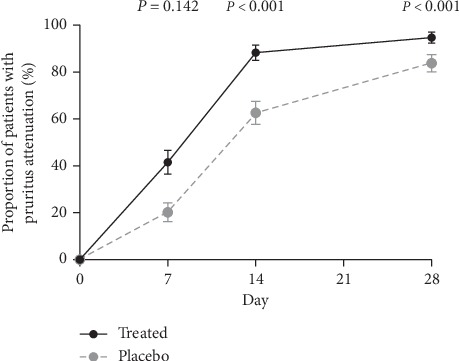
Reduction in pruritus. A greater percentage of patients in the OCG-treated group achieved pruritus attenuation than in the placebo group.

We also examined changes in the pruritus of patients. The proportion of patients with reduced pruritus was significantly higher in the OCG group versus placebo (day 7: 41.5% vs. 20.2%, *χ*^2^ = 10.295, *P*=0.001; day 14: 88.3% vs. 62.6%, *χ*^2^ = 17.007, *P* < 0.001; day 28: 94.7% vs. 83.8%, *χ*^2^ = 5.846, *P*=0.016, Figure 5).

### 3.3. Safety and Recurrence

Adverse events involved both local and systemic reactions, including local edema of both lower limbs, hypokalemia, transaminase elevation, hematuria, blood pressure elevation, and renal contusion (Table 3). Adverse events occurred in 11 patients, eight in the OCG group and three in the placebo group which was no statistically significant incidence rate (8.5% vs. 5.7%, *P* > 0.05). Two participants from the OCG group voluntarily withdrew the study. Of the eight patients experiencing adverse events in the OCG group, six were induced by the trial medications (Table 4). Of the three patients experiencing adverse events in the placebo group, only one was associated with trial medications. One patient (9.1%) from the treated group also experienced moderate edema of both lower limbs (Table 4).

A total of 15 patients developed recurrence, including three (3/94, 3.19%) from the OCG group and 12 (12/99, 12.12%) from the placebo group (*P*=0.021). Three cases of recurrence (two and one from the OCG and placebo groups, respectively) occurred in the first week after the end of the treatment, along with eight from the placebo group in the second week, and four (one from the OCG group and three from the placebo group) in the 4^th^ week.

## 4. Discussion

Traditional Chinese medicine (TCM) has been widely accepted in the treatment of eczema for more than 20 years [19]. This multicenter, randomized, double-blinded, placebo-controlled study showed that 4-week OGG combined with topical cream mometasone furoate 0.1% ointment resulted in statistically significantly higher proportions of patients achieving EASI reduction (71.73%) than the placebo combined with the topical cream (64.16%) for patients with moderate or worse chronic localized eczema. The EASI-50 reached 96.8% and EASI-75 was 47.9% on day 28 in the OCG group. Another indicator of therapeutic effects is IGA score. Consistent with EASI reduction, IGA score reductions were also significantly greater in the OCG group compared to placebo.

We found that OCG capsules relieved pruritus beginning on day 7 of the treatment. The early disruption of the itch-scratch cycle can reduce the risk of infection and scarring [20]. In addition, the recurrent rate was less in the OCG group. It is considered that OCG may regulate the systemic immune function in eczema patients.

Similar to our results, Fang et al. found that oral glycyrrhizin acid glycoside tablets combined with TCS showed better efficacy than the topical therapy alone in patients with chronic eczema in a randomized control trial; however, it is a single-center study used a single-blind study design [20]. Compound glycyrrhizin capsules also show good efficacy in the treatment of other inflammatory skin diseases, such as alopecia areata, psoriasis, and vitiligo [13, 14]. In China, compound glycyrrhizin is often used in an attempt to avoid the use of steroids, as the molecular structure of glycyrrhizin is similar to adrenal cortex hormones, yet causes no corticosteroid-associated adverse side effects [20]. Thus, OCG is a promising option for patients with steroid hormonal therapy contraindications, such as diabetes and hypertension.

The main component of compound glycyrrhizin capsules is glycyrrhizin acid, a pentacyclic triterpenoid and bioactive constituent of *Glycyrrhiza glabra* [21]. Glycyrrhizin acid has many pharmacological functions, including anti-inflammatory, antimicrobial, antioxidant, and antiaging. The anti-inflammatory mechanism of glycyrrhizin is associated with blockade of arachidonic acid metabolism and suppression of the classical complement pathway [22]. Compound glycyrrhizin can also reduce hydrocortisone metabolism and extend the turnover time of hydrocortisone in the body, thereby enhancing the therapeutic effects of hydrocortisone [23, 24]. The etiology and pathophysiology of eczema are complex, including genetics factors, barrier dysfunction, immune imbalance, and environmental factors. The therapeutic effect of glycyrrhizin on chronic eczema is considered through its anti-inflammatory mechanism.

In terms of safety, we found that OCG capsules showed a satisfactory safety profile for patients with chronic eczema, as no patients experienced serious adverse events during our study. Nevertheless, long-term use of OCG may increase the risk of some adverse events, such as pseudoaldosteronism [25, 26]. Known effects of pseudoaldosteronism were observed in our study, as three (3.2%) patients developed lower extremity edema, one (1.1%) experienced hypokalemia, and two (2.1%) had new-onset high blood pressure. All these patients were greater than 60 years old. Thus, the application of OCG capsules may increase the risk of pseudoaldosteronism in elderly patients.

We acknowledge several limitations of our study. First, this trial only included patients with chronic eczema and the efficacy of OCG capsules in acute and subacute eczema is still unknown. Second, our study population only included Chinese adults aged 18 to 80 years, limiting the generalizability of the findings to other patient populations. Thirdly, the follow-up duration of four weeks is a relatively short timeframe for observing eczema recurrence.

## 5. Conclusions

OCG combined with TCS demonstrated a favorable safety profile and satisfactory efficacy in the treatment of adults with chronic eczema. The combination therapy significantly improved the appearance of skin lesions, alleviated pruritus, and reduced disease recurrence. Thus, OCG may be a nonsteroidal agent with an additional effect for the treatment of chronic eczema by TCS.

## Figures and Tables

**Table 1:** Patient visit schedule and clinical tasks.

Items	Day 0	Day 7±1	Day 14 ± 2	Day 28 ± 3	Day 56 ± 3
	Enrollment	Interview 1	Interview 2	Interview 3	Follow-up
Informed consent form	×				
Inclusion/exclusion criteria	×				
Demographic data	×				
Previous and accompanying disease history	×				
Physical examination/blood pressure	×	×	×	×	
Blood, urine routine/liver and kidney function/electrolyte detection	×			×	
Electrocardiogram (ECG)	×			×	
Pregnancy screening (female)					
Disease assessment (signs and symptoms)	×	×	×	×	×
Drug distribution	×	×	×		
Drug recovery		×	×	×	
EASI score	×	×	×	×	
IGA score	×	×	×	×	
Itch symptom evaluation (VAS)	×	×	×	×	
Combined medication record	×	×	×	×	
Adverse event follow-up and records	×	×	×	×	

**Table 2:** Baseline patient demographics (FAS).

Characteristic	Total *N* = 193	Control *N* = 99	Treated *N* = 94	*t*/*χ*^*2*^	*P*
*Age (y)*					
Mean (SD)	43.64 (13.89)	42.25 (14.33)	45.10 (13.32)	1.43	0.16
Range, min-max (y)	18–73	18–69	22–73		

*Age distribution*					
18–39 y	70	29	41		
40–59 y	93	49	44		
≥60 y	30	16	14		
Sex, male, *n* (%)	71 (36.8%)	40 (40.4%)	31 (33.0%)	1.14	0.29
Height (cm), mean (SD)	165.07 (8.00)	165.29 (8.85)	164.85 (7.08)	−0.37	0.71
Weight (kg), mean (SD)	63.65(12.60)	63.06(9.75)	64.27(15.07)	0.67	0.51
BMI, mean (SD)	23.29 (3.96)	23.05 (2.88)	23.55 (4.84)	0.87	0.39

*Baseline IGA, n (%)*					
Moderate (Grade 3)	79 (40.9)	37 (39.4)	42(42.4)	0.187	0.665
Severe (Grade 4)	114(59.1)	57(60.6)	57(60.6)		

*BSA (%)*					
Mean (SD)	10.88 (2.54)	10.94 (2.75)	10.71 (2.01)	0.034	0.967
Range, min-max	6–14	6–13	5–14		

*EASI*					
Mean (SD)	10.29 (1.09)	10.35 (1.16)	10.23 (1.07)	0.72	0.452
Range, min-max		7.89–13.23	7.67–13.31		

BSA: body surface area; IGA: Investigator's Global Assessment score; EASI: Eczema Area and Severity Index.

**Table 3:** Adverse event incidence.

Adverse event *n* (%)	Total *N* = 193	Control *N* = 99	Treated *N* = 94
Edema of both lower limbs	3 (1.6)	0 (0)	3 (3.2)
Hypokalemia	1 (0.5)	0 (0)	1 (1.1)
Facial edema	1 (0.5)	1 (1.0)	0 (0)
Hematuria	1 (0.5)	1 (1.0)	0 (0)
Trunk and limb pruritus	1 (0.5)	0 (0)	1 (1.1)
Renal contusion	1 (0.5)	0 (0)	1 (1.1)
Blood pressure elevation	2 (1.0)	0 (0)	2 (2.1)
Transaminase elevation	1 (0.5)	1 (1.0)	0 (0)

Adverse events include abnormal clinical laboratory test results, physical examination, and physiological test results.

**Table 4:** Summary of subjects experiencing adverse events.

Clinical center	Subject number	Adverse event	Discontinued trial	Severity	Correlation	Induced by test drug
*OCG* *+* *TCS group*						
H1	32	Edema of both lower limbs	No	Moderate	Possible	Yes
H1	34	Hypokalemia	No	Mild	Positive	Yes
H1	35	Edema of both lower limbs	Yes	Mild	Possible	Yes
H2	210	Persistent blood pressure elevation	No	Mild	Possible	No
H7	125	Trunk and limb skin itch	Yes	Mild	Possible	Yes
H2	184	Edema of both lower limbs	No	Mild	Possible	Yes
H2	190	Blood pressure elevation	No	Mild	Possible	Yes
H2	202	Renal contusion	No	Mild	Negative	No

*Placebo* *+* *TCS group*						
H2	203	Transaminase elevation	No	Mild	Negative	No
H4	95	Urine occult blood	No	Mild	Possible	No
H3	164	Facial edema	No	Mild	Possible	Yes

## Data Availability

The data used to support the findings of this study are currently under embargo while the research findings are commercialized. Requests for data, 6 months after publication of this article, will be considered by the corresponding author.

## Supplementary Materials

Supplementary Materials Supplementary Table 1: Eczema Area and Severity Index (EASI) score calculation and definitions. Supplementary Table 2: Investigator's Global Assessment (IGA) score definition. Supplementary Table 3: pruritus severity (VAS).
