# Supplementary material for: A Multicenter, Randomized, Double-Blind, Placebo-Controlled Study of Compound Glycyrrhizin Capsules Combined with a Topical Corticosteroid in Adults with Chronic Eczema
Source: Evid Based Complement Alternat Med. 2020 Mar 30;2020:6127327. doi: 10.1155/2020/6127327 (PMC7149328; doi:10.1155/2020/6127327)
Supplement: Supplementary Materials — Supplementary Table 1: Eczema Area and Severity Index (EASI) score calculation and definitions. Supplementary Table 2: Investigator's Global Assessment (IGA) score definition. Supplementary Table 3: pruritus severity (VAS). [file 6127327.f1.docx]

**Supplementary Tables**

**Supplementary Table 1 Eczema area and severity index (EASI) score calculation and definitions**

| **Score** | **Disease severity** | **Definition** |
| --- | --- | --- |
| Erythema (redness) |  |  |
| 0 | None | No redness |
| 1 | Mild | Mildly detectable erythema; pink |
| 2 | Moderate | Dull red; clearly distinguishable |
| 3 | Severe | Deep, dark red; marked and extensive |
| induration/papulation/edema | | |
| 0 | None | None |
| 1 | Mild | Slightly perceptible elevation |
| 2 | Moderate | Clearly perceptible elevation but not extensive |
| 3 | Severe | Marked and extensive elevation |
| Excoriations |  |  |
| 0 | None | No evidence of excoriation |
| 1 | Mild | Mild excoriation |
| 2 | Moderate | Definite excoriation |
| 3 | Severe | Marked, deep, or extensive excoriation |
| Lichenification |  |  |
| 0 | None | No epidermal thickening |
| 1 | Mild | Minor epidermal thickening |
| 2 | Moderate | Moderate epidermal thickening; accentuated skin lines |
| 3 | Severe | Severe epidermal thickening; deeply accentuated skin lines |

The shape of skin lesions was defined as erythema (E), induration/papulation (I), excoriation (Ex), and lichenification (L). Lesion area score 0, 1, 2, 3, 4, 5, and 6 represent 0%, < 10%, 10%-29%, 30%-49%, 50%-69%, 70%-89%, and 90%-100% of the total body surface area were affected by the skin lesions, respectively. Eczema area and severity index (EASI) = head/neck lesion area score × total head/neck severity score (E+I+Ex+L) × 0.1 + upper limb lesion area score × total upper limb lesion severity score (E+I+Ex+L) × 0.2 + trunk lesion area score × total trunk lesion severity score (E+I+Ex+L) × 0.3 + lower limb lesion area score × total lower limb lesion severity score (E+I+Ex+L) × 0.4. EASI was assessed at the screening/enrollment interview, days 7, 14, and 28 after the treatment. EASI ranges from none (0) to the maximally severe (72).

**Supplementary Table 2 Investigator’s Global Assessment score definition**

| **Score** | **Grade** | **Definition** |
| --- | --- | --- |
| 0 | Clear | no inflammatory sign |
| 1 | Almost clear | just visible red spot and papules/infiltration |
| 2 | Mild | Mild erythema and mild papules/infiltration |
| 3 | Moderate | moderate erythema and moderate papules/infiltration |
| 4 | Severe | severe erythema and severe papules/infiltration |
| 5 | very severe | Very severe erythema and very severe papules/infiltrates with exudate/scab |

To assess the overall disease severity, Investigator’s Global Assessment (IGA) was determined at the screening/enrollment interview and day 7, 14 and 28 after the treatment. IGA was scored from clear (0) to the very severe (5).

**Supplementary Table 3 Pruritus severity**

| Scale | Grade | Definition |
| --- | --- | --- |
| 0 | None | No itch |
| 1 | Mild | Slightly aware, easy to stand, no scratching |
| 2 | Moderate | Clearly aware, suffering but tolerable and scratching sometimes |
| 3 | Severe | hard to bear, affecting daily activities and sleep, and scratching often |

Pruritus severity was assessed at screening/enrollment interview, day 7, 14, and 28 after the treatment and scaled from none (0) to severe (3).
